# The role of vitamin C in the treatment of pain: new insights

**DOI:** 10.1186/s12967-017-1179-7

**Published:** 2017-04-14

**Authors:** Anitra C. Carr, Cate McCall

**Affiliations:** 1 Department of Pathology, University of Otago, Christchurch, PO Box 4345, Christchurch, 8140 New Zealand; 2 Centre for Postgraduate Nursing Studies, University of Otago, Christchurch, PO Box 4345, Christchurch, 8140 New Zealand

**Keywords:** Vitamin C, Chronic regional pain syndrome, Post-herpetic neuralgia, Cancer quality of life, Opioid requirements

## Abstract

The vitamin C deficiency disease scurvy is characterised by musculoskeletal pain and recent epidemiological evidence has indicated an association between suboptimal vitamin C status and spinal pain. Furthermore, accumulating evidence indicates that vitamin C administration can exhibit analgesic properties in some clinical conditions. The prevalence of hypovitaminosis C and vitamin C deficiency is high in various patient groups, such as surgical/trauma, infectious diseases and cancer patients. A number of recent clinical studies have shown that vitamin C administration to patients with chronic regional pain syndrome decreases their symptoms. Acute herpetic and post-herpetic neuralgia is also diminished with high dose vitamin C administration. Furthermore, cancer-related pain is decreased with high dose vitamin C, contributing to enhanced patient quality of life. A number of mechanisms have been proposed for vitamin C’s analgesic properties. Herein we propose a novel analgesic mechanism for vitamin C; as a cofactor for the biosynthesis of amidated opioid peptides. It is well established that vitamin C participates in the amidation of peptides, through acting as a cofactor for peptidyl-glycine α-amidating monooxygenase, the only enzyme known to amidate the carboxy terminal residue of neuropeptides and peptide hormones. Support for our proposed mechanism comes from studies which show a decreased requirement for opioid analgesics in surgical and cancer patients administered high dose vitamin C. Overall, vitamin C appears to be a safe and effective adjunctive therapy for acute and chronic pain relief in specific patient groups.

## Background

Pain is defined as ‘an unpleasant sensory and emotional experience associated with actual or potential tissue damage, or described in terms of such damage’ [1]. The taxonomy of pain has developed through the work of the International Association for the Study of Pain and encompasses broad classifications that relate to the aetiology of pain, such as nociceptive (pain in response to injury) and neuropathic (nerve pain or pain in response to nerve damage), as well as particular pain features, such as allodynia (increased sensitization of neurons) and hyperalgesia (increased sensitivity to pain). Time course influences, such as chronic and acute, are also taken into consideration. The principal organ of pain is the brain. Noxious stimuli, once transduced, are conducted as nociceptive signals to the central nervous system via the spinal cord and ascend to the higher centres. It is here that the experience of pain is perceived and experienced in a complex and dynamic interaction between cerebral areas both sophisticated and primal. Pain is a transdiagnostic symptom and while somatic pathology plays a role in activating pain pathways, psychosocial, cultural and environmental factors influence the experience of pain over time [2].

In the absence of empirical evidence to validate the presence of pain measurement relies largely on eliciting the experience of the patient through self-report. It is understood that pain is an individual and subjective experience and may or may not be associated with evident tissue damage or disease. Furthermore, there are many influencing factors, such as mental state (both organic and psychological), coping strategies, social/cultural context, experience, and co-symptoms. The patient self-report can be validated using multiple outcome measurement tools designed to capture the complexity of the pain experience, for example, the visual analogue and numerical pain rating scales [3], the McGill pain questionnaire [4], and the Brief Pain Inventory [5].

Recent epidemiological evidence has indicated an association between spinal pain and suboptimal vitamin C status [6]. Musculoskeletal pain is also a symptom of the vitamin C deficiency disease scurvy [7]. Furthermore, accumulating evidence indicates that vitamin C administration can exhibit analgesic properties in some clinical conditions. In this review we focus on human studies investigating the role of vitamin C in orthopedic, virus-associated, cancer-related, and post-surgical pain. Preclinical models of pain are not always directly comparable to clinical scenarios of pain [8]. Nevertheless, we discuss some preclinical studies, although these have been carried out in animals that can synthesise their own vitamin C and, as such, are not ideal models for the human vitamin C-requiring situation. Vitamin C has a number of important functions in the body, primarily through acting as a cofactor for a family of biosynthetic and regulatory metallo-enzymes. These functions include synthesis of neurotransmitters and peptide hormones, and regulation of transcription factors and gene expression [9, 10]. We cover the potential analgesic mechanisms of vitamin C and propose a novel analgesic mechanism involving the biosynthesis of amidated opioid peptides. We also discuss study limitations, highlighting the need for an improved understanding of the pharmacokinetics of oral and intravenous vitamin C in future studies.

### Vitamin C deficiency and pain

Pain is a symptom of the vitamin C deficiency disease scurvy, presenting primarily within the musculoskeletal system as arthralgia in the knees, ankles and wrists, as well as myalgia [7, 11]. Children in particular suffer from severe lower limb pain, as evidenced by numerous case reports in the literature [12–16]. There have also been reports of adults and the elderly experiencing musculoskeletal pain due to severe vitamin C deficiency [17, 18]. Scurvy-related pain appears to be primarily due to bleeding into the musculoskeletal tissues, which can become so debilitating that patients are unable to walk [7]. Bleeding into the muscles and other soft tissues results in swelling and tenderness in the affected area, whilst bleeding into the hip, knee and ankle joints results in hemarthroses, and bleeding into the periosteum results in severe bone pain. Pain due to vitamin C deficiency can be completely resolved within a week or two following supplementation with intakes of vitamin C that will eventually result in plasma saturation (i.e. ≥200 mg/day, see examples cited in [12]).

It is interesting to note that Duggan et al. reported that a child’s painful scurvy symptoms began after an upper respiratory infection and that “possibly the increased metabolic needs associated with this infection unmasked a subclinical vitamin C deficiency [14].” Khalid also reported three cases of children suffering from respiratory infections or gastrointestinal dysfunction who concurrently developed painful swellings of their joints [16]. The author stated that “scurvy occurred as a result of their increased requirement of vitamin C due to stress of illness combined with poor dietary intake. It is therefore recommended that during illness one should be careful about the intake of vitamin C, keeping in mind that acute illness rapidly depletes stores of ascorbic acid. Those already malnourished are more prone to this development [14].” Similarly, others have reported painful scurvy symptoms following confirmed or suspected respiratory infection [18, 19], stating that “sepsis of either digestive or pulmonary origin, leading to sustained metabolic demand, might have acted as a precipitating factor [18].” As such, it is possible that other hospital-associated pain may be partly due to vitamin C deficiency, which is relatively prevalent in hospital settings [20–23].

### Vitamin C deficiency and enhanced requirements in patients

Vitamin C deficiency (defined as plasma vitamin C concentrations <11 µmol/L) is relatively rare in the general population of developed countries, with a prevalence of 6% reported in the United States [24]. However, vitamin C deficiency and scurvy has been reported to occur in elderly hospitalized patients [25, 26], critically ill patients [18, 27, 28], and cancer patients [29]. Hospitalized patients, in general, are more likely to present with hypovitaminosis C (defined as plasma vitamin C concentrations <23 µmol/L), and a higher proportion of hospital patients exhibit deficiency compared with the general population [20, 21]. Trauma and surgery are known to significantly deplete vitamin C concentrations [22], and patients with severe infections and sepsis also have significant depletion of vitamin C [23]. Cancer patients typically have lower vitamin C status than healthy controls [30, 31], with a large proportion of them presenting with hypovitaminosis C and outright deficiency [32].

It is interesting to note that animals, which can synthesise their own vitamin C, will increase their synthesis of the vitamin if they become stressed, are under a disease burden, or are administered drugs, including analgesics [33–35]. Therefore, it seems likely that hospitalised patients, who are under enhanced physiological stress, often presenting with a disease burden, and being administered multiple drugs, will have enhanced requirements for vitamin C. In support of this premise, vitamin C intakes of 100–200 mg/day provide adequate to saturating plasma status in healthy individuals [36], however, much higher gram doses are required to normalize plasma vitamin C status in surgical and critically ill patients [22, 23]. Administration of vitamin C to cancer patients results in lower plasma concentrations compared with healthy controls [37], suggesting a depleted body pool. Furthermore, administration of some anti-cancer therapies has been shown to significantly decrease patient vitamin C concentrations and scurvy-like symptoms have been reported [38–40]. Other drugs, such as aspirin, may also interfere with vitamin C uptake and could potentially result in hypovitaminosis C in individuals with low vitamin C intake [41]. Overall, these studies indicate an increased utilisation of and requirement for vitamin C in different patient cohorts.

### Vitamin C and orthopedic pain

Persistent musculoskeletal pain and associated complex regional pain syndrome (CRPS) present particular features underpinned by complex dynamic neural plasticity [3]. Features such as allodynia and hyperalgesia allude to sensitization of the nociceptive neurons, both peripheral and central, which invokes a cascade of effects experienced as pain that is both difficult to predict and manage. Vitamin C deficiency has been associated with spinal pain, primarily neck, lower back and arthritis/rheumatism [6]. The vitamin has been shown to exert a number of regulatory effects on cells of the skeletal system, including osteogenic, chondrogenic and osteoblastogenic [42]. Mechanisms of vitamin C action in bone cells primarily involve up- or downregulation of the expression of specific genes through regulation of transcription factors and epigenetic marks.

A number of randomized controlled trials have investigated the effect of vitamin C supplementation on the incidence of CRPS in wrist and ankle surgery patients (Table 1) [43–47]. Doses of vitamin C used in these studies ranged from 0.2 to 1.5 g/day for 45–50 days post-surgery. All studies, but one [43], showed a decreased incidence of CRPS in the patients receiving vitamin C, with vitamin C doses ≥0.5 g/day being the most efficacious [44]. Previous research has indicated that surgical patients have high vitamin C requirements and supplementation with >0.5 g/day vitamin C is required to restore normal vitamin C status in these patients [22]. The results of these studies have been pooled in various combinations in a number of recent meta-analyses [48–52] and all, but one [50], concluded that the evidence indicates that daily administration of vitamin C can decrease the incidence of CRPS following distal fracture surgery.

**Table 1:** The effect of vitamin C on complex regional pain syndrome (CRPS) and other orthopedic pain

Study type	Intervention	Findings
Placebo controlled RCT		
Wrist fractures [43]^a^	i. Placebo (N = 167)	i. 20–42% CRPS (at 6 weeks), 5–16% CRPS (at 1 year)
	ii. 500 mg/day oral vitamin C (N = 169) for 50 days	ii. 40–42% CRPS (at 6 weeks), 6–16% CRPS (at 1 year)
Wrist fractures [44]^a^	i. Placebo (N = 99)	i. 10% CRPS
	ii. 200 mg/day oral vitamin C (N = 96)	ii. 4% CRPS
	iii. 500 mg/day oral vitamin C (N = 144)	iii. 2% CRPS*
	iv. 1.5 g/day oral vitamin C (N = 118) for 50 days	iv. 2% CRPS*
Wrist fractures [45]^a^	i. Placebo (N = 63)	i. 22% CRPS
	ii. 500 mg/day oral vitamin C (N = 52) for 50 days	ii. 7% CRPS* (at 1 year follow up)
Hip/knee osteoarthritis [55]	Placebo or 1 g/day oral vitamin C (N = 133) Cross-over design, 14 days with 7 day washout	5% ↓ pain (VAS)*
Controlled prospective		
Foot and ankle surgery [46]^a^	i. Control (N = 235)	i. 10% CRPS
	ii. 1 g/day oral vitamin C (N = 185) for 45 days	ii. 2% CRPS*
Wrist fracture surgery [47]^a^	i. Control (N = 100)	i. 10% CRPS
	ii. 1 g/day oral vitamin C (N = 95) for 45 days	ii. 2% CRPS* (at 90 day follow up)
Paget’s disease of bone [59]	i. Calcitonin (N = 13)	i. Pain relief in 85%, marked ↓ pain in 31%
	ii. Calcitonin + 3 g/day vitamin C (N = 11) for 2 weeks	ii. Pain relief in 73%, marked ↓ pain in 45%
Uncontrolled prospective		
Arthritic joint replacement surgery [54]^a^	500 mg/day oral vitamin C (N = 34) for 50 days	0% CRPS cases
Paget’s disease of bone [58]	3 g/day oral vitamin C (N = 16) for 2 weeks	↓ Pain in 50%, no pain in 20% (within 5-7 days)
Case report		
Rheumatoid arthritis [56]	50 g IV vitamin C twice/week for 4 weeks	Before: 100% pain (QLQ)
		After: 0% pain

*IV* intravenous, *VAS* visual analog scale, *QLQ* quality of life questionnaire

* *P* < 0.05

^a^Study was included in CRPS meta-analysis [48–52]

Patients undergoing joint replacement surgery for osteoarthritis were administered 0.5 g/day prophylactic vitamin C for 50 days post-surgery (Table 1) [53, 54]. Although osteoarthritis of the carpometacarpo joint can be complicated by CRPS, no cases of CRPS were observed under vitamin C prophylaxis. A randomized placebo-controlled crossover trial carried out with 133 patients with osteoarthritis of the hip or knee joint showed reduced pain following consumption of 1 g/day calcium ascorbate for 2 weeks as determined by the visual analogue scale (*P* < 0.008) [55]. The observed decrease in pain was less than half that reported for non-steroidal anti-inflammatories. We have shown a complete decrease in pain in a patient with rheumatoid arthritis following administration of twice weekly infusions of high-dose vitamin C [56]. This data suggests that vitamin C may be more effective for the pain associated with rheumatoid arthritis than osteoarthritis, or that intravenous administration of the vitamin may be more effective than oral administration in patients with arthritis. It is noteworthy that the average vitamin C status of patients with rheumatoid arthritis is less than half that of healthy controls (i.e. 27 ± 13 versus 70 ± 21 µmol/L, respectively) [57].

Paget’s disease of bone is a chronic disorder caused by the excessive breakdown and formation of bone and disorganized bone remodeling which results in bone weakening, misshapen bones, fractures, arthritis, and pain. An early study in 16 patients with Paget’s disease of bone showed that oral doses of 3 g/day vitamin C for 2 weeks decreased pain in 50% of the patients and resulted in a complete elimination of pain in 20% of the patients [58]. Excretion of hydroxyproline was elevated following administration of vitamin C, and was highest in those patients who experienced complete relief of pain. This suggests that vitamin C is acting as a cofactor for the hydroxylase enzymes responsible for collagen synthesis [10]. When 3 g/day vitamin C was administered to Piaget’s patients in combination with normal calcitonin treatment, there was no additional attenuation of pain above calcitonin alone, although normalization of hydroxyproline excretion was observed, in contrast to calcitonin treatment, which decreases hydroxyproline excretion [59].

### Vitamin C and virus-associated pain

Infection with viral pathogens is commonly associated with myalgia, arthralgia or neuralgia [60]. Herpes zoster infection (shingles) results in a painful skin rash which generally lasts 2–4 weeks. However, some people develop ongoing nerve pain, a condition known as postherpetic neuralgia, which may last for months or years and is due to nerve damage or alterations caused by the virus in discrete dermatomes. Pain can be mild to extreme in the affected dermatome, and can include sensations of burning pain, itching, hyperesthesia (oversensitivity), or paresthesia (tingling, pricking, or numbness, ‘pins and needles’) [61, 62]. Analysis of the nutrient status of 50 patients with postherpetic neuralgia indicated significantly lower circulating concentrations of vitamin C compared with 50 healthy controls (i.e. 30 ± 21 versus 76 ± 31 µmol/L, respectively) [63]. More than 50% of the patients had hypovitaminosis C (i.e. <23 µmol/L) and vitamin C concentrations ≤45 µmol/L were found to independently increase the risk of post-herpetic neuralgia (adjusted OR 21; 95% CI 6, 76; *P* < 0.001).

A number of case studies have indicated that both acute and postherpetic neuralgia can be dramatically decreased following intravenous vitamin C infusions (2.5–15 g daily or every other day for 5–14 days) [64–67]. In an uncontrolled follow-up study, Schencking et al. recruited 64 patients with Herpes Zoster who were subsequently administered 7.5 g intravenous vitamin C two to four times a week for a total of 2 weeks [68]. Baseline pain was reported to be 58% (as determined by VAS), which decreased to 22% within 2 weeks and this had decreased to 6% at 12 week follow-up. Overall, there was a decrease in pain for 92% of the patients. The lack of a control group is a major limitation of this study.

Two placebo-controlled trials have investigated the effect of intravenous vitamin C on acute and post-herpetic neuralgia (Table 2) [69, 70]. Chen et al. carried out a trial in 41 patients with postherpetic neuralgia randomized to receive intravenously 50 mg vitamin C/kg body weight three times over 5 days, or placebo infusion [69]. Patients receiving vitamin C reported a larger decrease in numeric rating scale for pain, and a greater global impression of change. Another recent RCT in 87 herpes zoster patients, randomized to receive 5 g intravenous vitamin C or placebo three times over 5 days, found no effect on acute pain within the first 4 weeks of hospitalization, but did show a decreased incidence of postherpetic neuralgia and significantly decreased pain at 8 and 16 weeks follow up [70].

**Table 2:** The effect of vitamin C on acute and chronic viral-associated pain

Study type	Intervention	Findings
Placebo controlled RCT		
Herpes Zoster [70]	i. Placebo infusion (N = 42)	i. 4.2 ↓ VAS, 57% PHN incidence
	ii. 5 g IV vitamin C (N = 45) on days 1, 3, 5	ii. ≥5.6 ↓ VAS*, 31% PHN incidence* (at 8 and 16 week follow up)
Postherpetic neuralgia [69]	i. Placebo infusion (N = 20)	i. 0.9 ↓ NRS, 10% PGIC
	ii. 50 mg IV vitamin C/kg body weight (max dose 2.5 g/day) (N = 21) three times over 5 days	ii. 3.1 ↓ NRS*, 62% PGIC* (at 7 day follow up)
Uncontrolled prospective		
Herpes Zoster [68]	7.5 g IV vitamin C (N = 64) 2–4 times/week for 2 weeks	Baseline: 58% pain (VAS)
		Week 2: 22% pain
		Week 12: 6% pain
Chikungunya virus—moderate to severe pain [75]	H_2_O_2_ + 25–50 g IV vitamin C (N = 56) single infusion	Before: 80% pain (NRS)
		After: 20% pain, no pain in 9% of patients
Case report		
Parvovirus B19 viremia—chronic arthralgia [74]	i. 10 g/day oral vitamin C for 10 days	i. Before: 30% pain (VAS) After: 5% pain
	ii. 10 g/day oral vitamin C for 3 weeks	ii. Before: 40% pain (VAS) After: 10% pain (at 3–5 week follow up, there was↓ pain within 5 days)
Chikungunya virus—severe joint pain [73]	100 g/day IV vitamin C for 2 days	Pain resolved within 24 h
Refractory herpes zoster-associated pain [67]	4 g/day IV vitamin C for 5 days	Before: 70% pain (VAS)
		After: 0% pain (at 3 month follow up)
Herpes zoster—severe dermatological pain [66]	Cantharidin + 7.5 g IV vitamin C every 2 days for 2 weeks	Before: 80% pain (NAS)
		After: 40% pain (within 2 weeks), 0% pain (at 8 week follow up)
Acute herpetic neuralgia [65]	15 g IV vitamin C every 2 days for 12 days	Before: 80% pain (VAS)
		After: 0% pain (within 8 days)
Acute herpetic neuralgia [65]	15 g IV vitamin C every 2 days for 16 days	Before: 100% pain (VAS)
		After: 0% pain (within 12 days)
Postherpetic neuralgia [64]	2.5 g IV vitamin C every 2 days for 5 days	Before: 73% pain (NRS)
		After: 0% pain (within 7 days and at 3 month follow up)

*IV* intravenous, *NAS* numerical analogue scale, *NRS* numeric rating scale, *PCIG* patient global impression of change, *PHN* postherpetic neuralgia, *VAS* visual analogue scale

* *P* < 0.05

Chikunguya virus infection is characterized by severe joint pain, which typically lasts weeks or months, and sometimes years [71]. Parvovirus B19 infection (also known as fifth disease) may also present with acute or persistent arthropathy, painful swelling of the joints that feels similar to arthritis [72]. Two cases of severe arthralgia associated with Chikungunya and parovirus B19 reportedly responded to high dose oral (10 g/day) and intravenous vitamin C treatments (Table 2) [73, 74]. Despite one case having 100 g/day vitamin C infusions, no adverse side effects were reported [73]. An uncontrolled prospective study carried out in 56 patients with Chikungunya virus indicated that a single infusion of 25–50 g intravenous vitamin C (administered with a 3% hydrogen peroxide solution) provided a 60% decrease in pain and completely eliminated pain in 9% of the patients [75].

### Vitamin C and cancer-related pain

Pain is one of the most common symptoms reported by cancer patients, and can seriously affect their quality of life [76]. Pain associated with cancer can be related to the primary tumour, cancer treatment, associated procedures and as a consequences of disease progression and metastasis. Furthermore, cancer pain may include several types of pain and pain features occurring concurrently as mixed pain, such as nociceptive, neuropathic, and bone pain [3]. Cancer-associated pain resulting from metastasis to bone is a severe and complex condition comprising neuropathic, nociceptive and inflammatory pain [77, 78]. As mentioned above, cancer patients typically have depleted vitamin C status [30–32] as well as higher requirements than healthy controls [37], which could potentially be exacerbated by anti-cancer therapies [38–40].

High dose oral and intravenous vitamin C has been administered to cancer patients for many decades as a complementary and alternative therapy [79]. Although the efficacy of vitamin C as a cancer treatment is questionable, recent research has indicated a positive impact of high dose vitamin C on cancer- and chemotherapy-related quality of life, including pain [80]. Early studies of high dose vitamin C in patients with advanced cancer indicated that many patients experienced some improvement in subjective symptoms, including decreased pain and the need for analgesics [81, 82]. Cameron and Campbell [81] reported a number of cases of dramatic to complete amelioration of bone pain in patients with severe cancer-related pain given both high dose oral and intravenous vitamin C (Table 3). Retrospective studies of patients with bone metastases receiving 2.5 g intravenous vitamin C once weekly or during intensifying pain reported a range of responses, including 0–100% decreases in pain [83, 84]. These, and the earlier case studies [81], indicate that vitamin C can potentially provide dramatic improvements in pain relief in cancer patients with bone metastases.

**Table 3:** The effect of vitamin C on cancer-related pain

Study type	Intervention	Findings
Uncontrolled prospective		
Advanced cancer [90]	0.8–3 g IV vitamin C/kg body weight (N = 17) 4 days/week for 4 weeks	Before: 36% pain (N = 17) Week 1: 35% pain (N = 16) Week 2: 35% pain (N = 12) Week 3: 29% pain (N = 7) Week 4: 0% pain (N = 2) (EORTC QLQ)
Advanced cancer [89]	25–100 g IV vitamin C (N = 60) twice weekly for 4 weeks	Before: 18% pain Week 2: 14% pain Week 4: 10% pain (EORTC QLQ)
Terminal cancer [88]	10 g IV vitamin C (N = 39) twice over 1 week 4 g/day oral vitamin C for 1 week	Before: 30% pain Week 1: 21% pain (EORTC QLQ)
Controlled retrospective		
Bone metastases [84]	i. Control (N = 9)	i. ↑ pain (VAS)
	ii. Chemotherapy (N = 15)	ii. 0–80% ↓ pain
	iii. 2.5 g IV vitamin C (N = 15) during pain	iii. 0–100% ↓ pain, mean 50% ↓ pain
Breast cancer [87]	i. Control (N = 72)	i. 15% pain
	ii. 7.5 g IV vitamin C (N = 53) once weekly for ≥ 4 weeks	ii. 10% pain* (intensity of complaints during adjuvant therapy)
Uncontrolled retrospective		
Bone metastases [83]	2.5 g IV vitamin C (N = 11) once weekly for 3–10 weeks	0–100% ↓ pain (VAS), mean 49% ↓ pain
Case report		
Breast cancer [133]	50 g IV vitamin C twice weekly for 4 weeks	Before: 17% pain After: 8% pain (EORTC QLQ)
Terminal cancer [95]	30 g/day IV vitamin C for 1 week	Before: 17% pain After: 0% pain (EORTC QLQ)
Metastatic breast cancer [81]	10 g/day oral vitamin C for 550 days	Pain relief for >1 year
Breast cancer with skeletal metastases—severe pain [81]	5 g/day IV vitamin C for 7 days 8 g/day oral vitamin C for 70 days	Complete ↓ bone pain from day 4
Bladder cancer with skeletal metastases—intense pain [81]	10 g/day IV vitamin C for 10 days 10 g/day oral vitamin C for 24 days	Dramatic ↓ bone pain
Breast cancer with osteolytic metastases—severe bone pain [81]	10 g/day IV vitamin C for 7 days 10 g/day oral vitamin C for 27 days	Complete ↓ bone pain

*EORTC QLQ* European Organisation for the Research and Treatment of Cancer Quality of Life Questionnaire, *IV* intravenous, *VAS* visual analogue scale

* *P* < 0.05

Over the last decade a number of studies have attempted to quantify the effect of high dose vitamin C on cancer-related symptoms such as pain (Table 3). These studies have typically used the European Organisation for the Research and Treatment of Cancer Quality of Life Questionnaire (EORTC QLQ) [85]. The EORTC QLQ assesses the typical cancer-related symptoms of pain, fatigue, nausea/vomiting, dyspnea, appetite loss, sleep disturbance, constipation, and diarrhea using a 4 point Likert scale. A difference of 10–20% represents a medium change in quality of life [86]. Most quality of life studies have reported decreases of >30% pain as assessed by the EORTC pain scale in patients with cancer receiving intravenous vitamin C (Table 3). A retrospective study of patients with breast cancer receiving 7.5 g intravenous vitamin C once a week showed decreases in a number of cancer-associated symptoms using a 3 point Likert scale, including a 30% decrease in pain during adjuvant therapy in the vitamin C group compared with the control group [87].

Two prospective studies of patients with advanced cancer who were administered intravenous vitamin C at doses of 10–100 g vitamin C (twice a week) have shown 30–44% decreases in pain using the EORTC pain scale within 1–4 weeks [88, 89]. Yeom et al. [88] recruited 39 patients with terminal cancer who subsequently received 10 g intravenous vitamin C twice weekly for 1 week, followed by 4 g/day oral vitamin C for 1 week. Patients exhibited 30% pain at baseline (as measured by the EORTC-QLQ) and this decreased by one-third following vitamin C infusion (*P* = 0.013). Takahashi et al. [89] recruited 60 patients with advanced cancer who received 25–100 g intravenous vitamin C twice weekly for 4 weeks. Baseline pain in this cohort was 18% and this decreased by 44% following vitamin C infusion (*P* < 0.05, using the EORTC-QLQ). A Phase I RCT designed to assess the safety, tolerability and pharmacokinetics of high dose intravenous vitamin C in patients with advanced cancer also assessed quality of life as a secondary outcome [90]. This showed a decrease in pain for the few patients who completed the EORTC-QLQ at 3 and 4 weeks follow-up (Table 3).

### Vitamin C and opioid analgesic requirements

The use of opioid analgesia is widely considered an essential component in the management of moderate to severe pain, however, opioid use is associated with a well-documented side effect profile. Opioid effects, both therapeutic and adverse, are dose dependent and subject to significant inter-individual variability with bearing on symptoms including nausea and vomiting, constipation, and sedation and respiratory depression [91]. Co-analgesic agents and interventions that are opioid sparing may improve the analgesic effect and reduce adverse effects.

Cancer-related pain is typically managed with opioids [92]. In the early 1970s Cameron and Pauling [93] described dramatic decreases in opiate dependence in five patients with advanced cancer following high dose vitamin C administration. These patients were in considerable pain due to skeletal metastases and were receiving large regular doses of opiate analgesics (morphine or diamorphine). Within five to seven days of commencing vitamin C, four of the five patients became completely free from pain, and the fifth required only mild analgesics [81]. Several of these cases are summarized in Table 4. Interestingly, none of the patients experienced any withdrawal symptoms despite having received opiate analgesia for periods of weeks or months, nor did they request that their opiate regime be continued. It is interesting to note that vitamin C (at a dose of 300 mg/kg body weight/day for 4 weeks) has been shown to dramatically decrease the major withdrawal symptoms of heroin addicts compared with a control group who were treated with conventional medication only [94]. A complete decrease in morphine requirement was also observed in a patient with terminal cancer undergoing 30 g/day vitamin C infusion for palliative care [95]. Murata et al. [82] reported a dose-dependent decrease in opioid requirement in patients with terminal cancer who received vitamin C. In those who received 0.5–3 g/day vitamin C, 50% of the patients required opioid drugs, whereas only 17% of those who received 5–30 g/day vitamin C required opioids, compared with 79% in the control group (Table 4). A recent study failed to confirm a decrease in opioid requirement in 17 patients with a range of malignancies [96], however, the study lasted for only 3 days and the vitamin C dose was lower than in studies that reported positive findings (Table 4).

**Table 4:** The effect of vitamin C on opioid analgesic requirements

Study type	Intervention	Findings
Placebo controlled RCT		
Laparoscopic colectomy—for colon cancer [97]	i. Placebo (N = 48)	i. 16 mg morphine at 2 h, frequency of rescue analgesia: 1.4
	ii. 50 mg IV vitamin C/kg body weight (N = 49) prior to surgery	ii. 14 mg morphine at 2 h*, frequency of rescue analgesia: 0.8*, ↓ pain at 2, 6, 24 h (NRS)*
Uvulopalatopharyngoplasty with tonsillectomy [98]	i. Placebo (N = 20)	i. 46 mg pethidine, first dose at 3 h, number of requests: 1.3
	ii. 3 g IV vitamin C (N = 20) 30 min into surgery	ii. 6 mg pethidine*, first dose at 12 h*, number of requests: 0.2*, ↓ pain at 6, 12, 24 h (VAS)*
Cholecystectomy [99]	i. Placebo (N = 40)	i. 23 mg morphine
	ii. 2 g oral vitamin C (N = 40) prior to surgery	ii. 16 mg morphine* (at 24 h follow up)
Uncontrolled prospective		
Range of malignancies [96]	2 g oral vitamin C (N = 17) for 3 days	Before: 360 mg/day opioids After: 390 mg/day opioids
Controlled retrospective		
Terminal cancer [82]	i. Control (N = 19)	i. 79% required narcotics
	ii. 0.5–3 g/day oral vitamin C (N = 6)	ii. 50% required narcotics
	iii. 5–30 g/day oral vitamin C (N = 6)	iii. 17% required narcotics
Case report		
Intolerable fibrosarcoma-related pain [81]	10 g/day vitamin C for 19 days	Better control of pain by opiates
Breast cancer with skeletal metastases—severe pain [81]	5 g/day IV vitamin C for 7 days	No further need for opiates (from day 4)
	8 g/day oral vitamin C for 70 days	
Bladder cancer with skeletal metastases—intense pain inadequately controlled by morphine [81]	10 g/day IV vitamin C for 10 days	No further need for opiates
	10 g/day oral vitamin C for 24 days	

*IV* intravenous, *NRS* numeric rating scale, *VAS* visual analogue scale

* *P* < 0.05

Three recent placebo-controlled trials have been carried out to investigate the effect of vitamin C on opioid requirement for postoperative pain, two using intravenous vitamin C [97, 98] and one using oral vitamin C [99]. In the most recent, 97 patients undergoing laparoscopic colectomy for colon cancer were randomized to receive intravenously 50 mg vitamin C per kg body weight or placebo infused immediately after induction of anaesthesia (Table 4). A decrease in postoperative morphine consumption was observed at 2 h (*P* < 0.05) in the vitamin C group, as well as a decreased frequency of rescue analgesia (*P* < 0.01), and decreased pain at 2, 6 and 24 h post-surgery as assessed by the numeric pain rating scale (*P* < 0.05). In the other study, 40 patients undergoing uvulopalatopharyngoplasty with tonsillectomy, which is normally associated with intense postoperative pain, were randomized to receive intravenously either 3 g vitamin C or placebo 30 min into the surgery (Table 4). A decrease in post-operative pethidine dose was recorded for the vitamin C group compared with the placebo group (5 vs 46 mg, *P* = 0.0001), as well as a delay in the time of first dose of pethidine use (12 vs 3 h, *P* = 0.003), and a decline in the total number of times pethidine requested was requested (0.2 vs 1.3 times, *P* = 0.001). Visual analogue scale scores were also lower in the vitamin C group at all time points assessed (recovery, 6, 12, 24 h, *P* = 0.001). Opioid-based analgesics are typically used for postoperative analgesia, however these may complicate care by causing excessive sedation and respiratory depression. In contrast, no side effects were observed with the vitamin C treatment.

In an earlier study, a single oral dose of 2 g vitamin C or placebo was given to 80 randomised cholecystectomy patients 1 h prior to anesthesia (Table 4). Postoperative morphine consumption and verbal numerical rating scale scores for incisional pain were recorded for 24 h. Morphine consumption was lower in the vitamin C group versus the placebo group (16 vs 23 mg, *P* = 0.02) and, despite the lower opioid usage in the vitamin C group, there was no difference in reported pain intensity or side effects between the two groups [99]. Although baseline plasma vitamin C concentrations were not determined, blood samples were collected approximately 1 h post-randomisation for vitamin C analysis. The placebo group had marginal vitamin C status (23 ± 17 µmol/L) and the vitamin C group had 57 ± 28 µmol/L, although this is possibly an underestimate as oral vitamin C uptake typically takes more than 1 h to peak [100].

Support for the opioid-sparing effects of vitamin C has come from murine studies. Co-administration of 1 g/kg vitamin C with morphine prevented the development of morphine tolerance and physical dependence in mice [101]. Intraperitoneal administration of 400 mg/kg vitamin C significantly decreased self-administration of morphine and withdrawal syndrome signs in rats [102]. Vitamin C itself was shown to have antinociceptive effects in mice (ED50 of 206 mg/kg). Furthermore, it exhibited not only additive effects, but also synergistic effects, in combination with the opioids morphine and tramadol [103]. Thus, vitamin C administration appears to have potential application as an adjunctive therapy to decrease opioid requirements and dependence.

### Vitamin C and pain study design limitations

A major limitation of many of the vitamin C and pain studies is inappropriate study design due to a general lack of understanding around the pharmacokinetics of vitamin C. Oral vitamin C is transported through the intestinal epithelium via sodium-dependent vitamin C transporters (SVCT-1) [104]. Levine and coworkers have shown that oral vitamin C uptake becomes less efficient as the dose increases due to saturation of the transporters. Although an oral dose of 200 mg vitamin C is completely absorbed, at doses of 500 mg and 1250 mg vitamin C, <75% and <50% of the vitamin dose is absorbed [36]. Furthermore, steady state plasma vitamin C concentrations rarely exceed 80 µmol/L due to rapid renal clearance. In contrast, intravenously administered vitamin C, which bypasses the intestinally regulated uptake of oral vitamin C, can provide plasma concentrations that are 250 fold higher [36]. However, it should be noted that because vitamin C has a short half-life in plasma of approximately 2 h [90], the high (millimolar) plasma concentrations provided by intravenous administration are relatively transient. Therefore, to maximise uptake and plasma concentrations of vitamin C, the chosen intravenous (or oral) dose should ideally be administered in several smaller doses over the day [100].

Few of the cited pain studies have measured vitamin C concentrations in their patients either before or after administration of the vitamin C intervention. Administration of vitamin C to patients who already have adequate vitamin C status (i.e. ≥50 µmol/L) is unlikely to have a significant effect and is a limitation of many previous vitamin C studies [105]. Although many patient cohorts are likely to have less than adequate vitamin C status (i.e. <50 µmol/L) and hypovitaminosis C (<23 µmol/L), baseline measures should still be collected to allow stratification and/or sub-group analysis of the patient cohorts. For example, we have shown that volunteers with marginal vitamin C status (hypovitaminosis C) have an attenuated response to recommended daily intakes of vitamin C (i.e. 50 mg/day), likely due to suboptimal tissue status, and as such need higher intakes to reach adequate plasma concentrations [106]. This phenomenon is likely to be even more pronounced in hospitalized patients due to increased metabolic demands for vitamin C due to surgery, trauma, infection or other disease processes. Both surgical and infectious disease patients have significantly lower than normal vitamin C status and much higher vitamin C concentrations (0.5–3 g/day) are required for restoration to normal status [22, 23]. Similar trends are observed with patients with cancer [37].

Although a number of placebo-controlled studies have been carried out, primarily for CRPS, postherpetic neuralgia and post-surgical pain, none of the cancer quality of life studies have included placebo controls (Table 3). As such, it is not possible to determine the relative contribution of the placebo effect in these studies, particularly as this effect tends to be more prevalent with subjective measures such as pain [107]. Finally, a major limitation of many vitamin C and pain studies is the lack of mechanistic underpinnings.

### Potential analgesic mechanisms of vitamin C

As yet, there is no consensus as to the analgesic mechanism(s) by which vitamin C could be acting. Oxidative stress and inflammation have been implicated in the sequelae of many pathologies, including arthritis, CRPS, infection, cancer and surgical trauma. Vitamin C is a potent antioxidant [108] which can scavenge a wide range of reactive oxygen species and, thus, is capable of protecting cells and tissues from oxidative damage [109]. Because of its well-known antioxidant properties, this is the mechanism by which vitamin C is often assumed to act in conditions where oxidative stress has been implicated. This is, however, an overly simplistic assumption due to the numerous enzymatic reactions in which vitamin C acts as a cofactor in the body [9]. Vitamin C also exhibits anti-inflammatory properties, providing marked decreases in markers of inflammation such as C-reactive protein and pro-inflammatory cytokines, e.g. tumor necrosis factor, interferon, and interleukins [110]. The biochemical mechanisms underlying vitamin C’s ability to decrease pro-inflammatory mediators are currently unknown.

Vitamin C has a well-established role as a cofactor for the synthesis of catecholamine neurotransmitters, and hence is involved in neuromodulation [111]. Vitamin C is a cofactor for the enzyme dopamine β-hydroxylase, which converts dopamine into norepinephrine [112, 113]. Vitamin C may also facilitate the synthesis of dopamine through recycling the cofactor tetrahydrobiopterin, which is required for optimal activity of the rate-limiting enzyme tyrosine hydroxylase [114]. A similar tetrahydrobiopterin recycling mechanism has been proposed for vitamin C in the biosynthesis of the monoamine neurotransmitter serotonin [115]. It is noteworthy that both serotonin and norepinephrine reuptake inhibitors show efficacy in control of pain [116]. Ascorbate-deficient animal models exhibit decreased norepinephrine concentrations compared with controls [117–119]. Thus, administration of vitamin C to depleted patients may enhance endogenous synthesis of these neurotransmitters which may in turn contribute to the vitamin C-dependent analgesia observed in some patients.

**Fig. 1:**
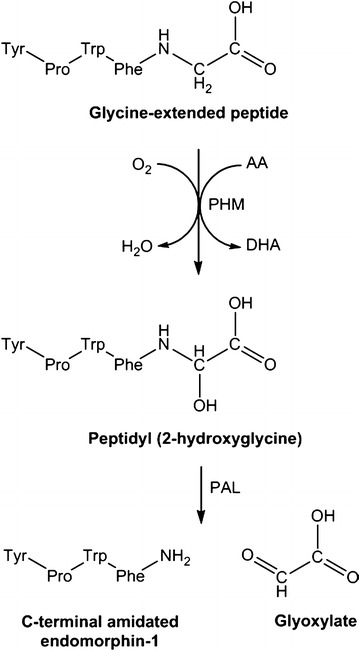
Proposed synthesis of endomorphin-1 by the vitamin C-dependent enzyme peptidylglycine α-amidating monooxygenase (PAM). The enzyme comprises a peptidylglycine α-hydroxylating monooxygenase (PHM) domain, which converts glycine-extended peptides into a hydroxyglycine intermediate, and a peptidyl α-hydroxyglycine α-amidating lyase (PAL) domain, which converts the hydroxyglycine intermediate into an amidated product. *AA* ascorbic acid, *DHA* dehydroascorbic acid

One currently unexplored analgesic mechanism involves the potential role of vitamin C in the synthesis of amidated opioid peptides. Vitamin C is a cofactor for the enzyme peptidylglycine α-amidating mono-oxygenase (PAM) [120]. PAM is the only known enzyme in humans capable of amidating the carboxy-terminus of peptide hormone precursors, a post-translational modification which is essential for their subsequent stability and/or biological activities [121]. A number of amidated neuropeptides have potent opioid activity. Endomorphin-1 and -2 are amidated tetrapeptides which have the highest known selectivity and affinity for the µ-opioid receptor of all known mammalian opioids [122]. Like other opioid peptides, it is presumed that the endomorphins are generated via post-translational cleavage of a larger precursor protein. For example, another amidated opioid peptide with analgesic properties, which was first identified in human adrenal medulla (adrenorphin or metorphamide) [123, 124], is derived from the proteolytic cleavage of proenkephalin A. A glycine-extended precursor of the opioid peptide would then act as the substrate for post-translational amidation by the ascorbate-dependent enzyme PAM to generate the active carboxy-amidated hormone (Fig. 1).

The endomorphins are widely expressed in the central nervous system and immune tissues [125]. They have well known analgesic properties, particularly for neuropathic pain, but also have anti-inflammatory activity, and have been proposed as potential therapeutic agents in the treatment of chronic inflammatory diseases such as rheumatoid arthritis and osteoarthritis [126]. As such, it is tempting to speculate that some of the observed anti-inflammatory effects of vitamin C could be due to enhanced synthesis of endomorphins. It is noteworthy that nervous and neuroendocrine tissues, where monoamine neurotransmitters and amidated neuropeptide hormones are synthesised, contain the highest concentrations of vitamin C in the body [127]. Depletion of amidated neuropeptide hormones has been demonstrated in humans during severe infection [128], which is known to significantly deplete vitamin C concentrations [23], and administration of vitamin C to animal models enhances the synthesis of these PAM-derived hormones [129]. Therefore, it is possible that depletion of vitamin C during acute or chronic disease or trauma could contribute to pain symptoms due to sub-optimal biosynthesis of analgesic neurotransmitters and neuropeptide hormones. The observation that vitamin C administration significantly decreases the requirement for opioid analgesics (Table 4) lends support to this hypothesis.

Calcitonin has been used for decades as a treatment for osteoporosis and other diseases involving accelerated bone turnover [130]. Calcitonin also has a direct analgesic effect on bone pain and has been utilised for improving the pain of acute vertebral fractures, malignant bone metastases, Paget’s disease, and complex regional pain syndrome [130]. It is interesting to note that calcitonin is an amidated peptide hormone, requiring post-translational amidation by PAM for full activity of the mature hormone [131]. Thus, vitamin C is likely to be also required as a cofactor for the synthesis of calcitonin. The analgesic properties of calcitonin appear to be independent of its effects on bone resorption and are possibly mediated through enhanced release of the potent analgesic β-endorphin [130]. Therefore, vitamin C may exhibit analgesia both indirectly, through calcitonin-dependent modulation of endorphins, and directly through enhanced synthesis of endomorphins.

## Conclusions

Acute and chronic pain can be debilitating for patients, particularly if not adequately managed by conventional analgesics. Accumulating evidence indicates that vitamin C can exhibit analgesic properties in some clinical conditions, thus potentially mitigating suffering and improving patient quality of life. Pain is costly because it requires medical treatment, complicates treatment of other conditions and results in lost productivity. In the USA the annual cost of pain was greater than the annual costs of heart disease, cancer, and diabetes [132]. Vitamin C is cost effective and appears to be a safe and effective adjunctive therapy for specific pain relief. Notably, it decreases the requirement for opioid analgesics, particularly post surgically and for bone metastasis, thus potentially diminishing the deleterious side effects of opioids. Future high quality studies are required to confirm these findings. Inclusion of placebo controls is preferred due to the subjective nature of pain, however, this can sometimes be difficult to justify in certain patient groups, hence the paucity of placebo-controlled trials for intravenous vitamin C and cancer quality of life. Ideally, studies should also include patients who have less than adequate vitamin C status at baseline (i.e. <50 µmol/L) to ensure that their concentrations are able to increase following supplementation. Overall, future studies should endeavor to ascertain the following aspects: measurement of vitamin C concentrations at baseline and following intervention to determine if specific patient groups respond, determination of the optimal rout of administration (i.e. enteral or parenteral), the optimal dose and frequency of vitamin C administration (which will likely differ depending upon the type of pain and associated conditions), and the potential mechanisms of action of vitamin C.

## Abbreviations

AA: ascorbic acid
CRPS: chronic regional pain syndrome
DHA: dehydroascorbic acid
EORTC: European Organisation for the Research and Treatment of Cancer
IV: intravenous
NAS: numerical analogue scale
NRS: numeric rating scale
PAL: peptidyl α-hydroxyglycine α-amidating lyase domain
PAM: peptidylglycine α-amidating mono-oxygenase
PCIG: patient global impression of change
PHM: peptidylglycine α-hydroxylating monooxygenase domain
PHN: postherpetic neuralgia
QLQ: quality of life questionnaire
RCT: randomized controlled trial
VAS: visual analog scale
